# Supplementary material for: CRISPR/Cas9-Mediated Disruption of the lef8 and lef9 to Inhibit Nucleopolyhedrovirus Replication in Silkworms
Source: Viruses. 2022 May 24;14(6):1119. doi: 10.3390/v14061119 (PMC9227026; doi:10.3390/v14061119)
Supplement: Supplementary file 1 [file viruses-14-01119-s001.zip › viruses-1709821-supplementary.pdf]

viruses

MDPI

Supplementary Material

Table S1. Primers used in this study.

Primer Name	Primer Sequence (5' to 3')
lef-8-F	TCAATTTTTCATTATCGTATTGTTGCAC
lef-8-R	ATGACGGACGTAGTTCAAGATTT
lef-9-F	ATGTTTTCTTTTTTGGATAAAACTCCTACT
lef-9-R	TCATTCAATAAACATGTCGAGCAAATG
lef8-sg1-F	GGAAGATTTAAATTCTGTGCGTTTTAGAGCTAGAAATAGCAAGTT
lef8-sg1-R	GCACAGAATTTAAATCTTCCACTTGTAGAGCACGATATTTTGTAT
lef8-sg2-F	CTCACTATAGGGCGAATTGGAGGTTATGTAGTACACATTGTTGTA
lef8-sg2-R	TTAAACACACCTTTCTGACCACTTGTAGAGCACGATATTTTGTAT
lef9-sg1-F	GGCGCTAACACAGACTGCGAGTTTTAGAGCTAGAAA-TAGCAAGTT
lef9-sg1-R	TCGCAGTCTGTGTTAGCGCCACTTGTAGAGCACGATATTTTGTAT
lef9-sg2-F	GCGCTATTACGGACGTGATGGTTTTAGAGCTAGAAA-TAGCAAGTT
lef9-sg2-R	CATCACGTCCGTAATAGCGCACTTGTAGAGCACGATATTTTGTAT
HindIII-F	CGAGGTCGACGGTATCGATAAGGTTATGTAGTACACATTGTTGTA
HindIII-R	TTTTCTTGTTATAGATATCAAAAAAAGCACCGACTCGGTGCCAC-TTTTTCAAGTTGATAAC-GGACTAGCCTTATTTTAACTTGCTATTTCTAGCTCTAAAAC
Overlap-F	GCTAGCCATTGACTCCGCGGATCGAAGGTTATGTAGTACACATT-GTTGTA
Overlap-R	CCGCGGAGTCAATGGCTAGCAAAAAAGCAC-CGACTCGGTGCCACTTTTTCAAGTTGATAAC-GGACTAGCCTTATTTTAACTTGCTATTTCTAGCTCTAAAAC
lef8-F-mut-test	CTTTCCGACATTCTGTTGCCT
lef8-R-mut-test	TGCGCTCTTAATGAACAGCTC
lef9-F-mut-test	CGTGTACACAGACACGTCAGCA
lef9-R-mut-test	GCTATAAGCTGTGTGAGCTGT
3'Invers F1	TAAAAAAAAACAAAAACTCAAAATTTCTTC
3'Invers R1	GTTTGTTGAATTTATTATTAGTATGTAAGT
3'Invers F2	CAAAACTTTTATCGAATTCCTGCAG
3'Invers F2	AACCTCGATATACAGACCGA
M13-F	GTAAAACGACGGCCAGT
M13-R	CAGGAAACAGCTATGAC
gp64-qPCR-F	CTTTAATGAGCAGACACGCAG
gp64-qPCR-R	GCGGCGTTTCTACTTCGAAA
lef3-qPCR-F	CGGAAGAGGTAGAACGGTCA
lef3-qPCR-R	GTGCGAGGCTAAAGAAAACG
ie-1-qRT-F	GCTCAAGACCACTGATAATCTC
ie1-qRT-R	AATCGTCCAAGTATTCGTCCA
p143-qRT-F	TGGCTTCATACTTTAGCAACC
p143-qRT-R	GTTTGACGATGACAACCACAG
vp39-qRT-F	TCTAAATCTCAATTCCTCCGTG
vp39-qRT-R	GCATTCTAGACACCACAAACC
p10-qRT-F	CCATTGCGGAAACTAACACA
p10-qRT-R	AGCAGTGTCACCGGTCAATA
BmRP49-qPCR-F	AAACATACAAGATGGCTATAAGACCTG
BmRP49-qPCR-R	TTTATAAATGACATGTGAACATACCTC
Pjet-F	CGACTCACTATAGGGAGAGCGGC
Pjet-R	AAGAACATCGATTTTCCATGGCAG
F3421	GTGGAGCTCCAGCTTTTGTT
R3667	GTGAGTCAAAATGACGCATG
